# Nursing Care during the Perioperative within the Surgical Context**

**DOI:** 10.17533/udea.iee.v40n2e02

**Published:** 2022-09-15

**Authors:** Ángela María Salazar Maya

**Affiliations:** 1 Nurse, Ph.D. Professor at Universidad de Antioquia and Universidad CES. Colombia. Email: angela.salazar@udea.edu.co Universidad de Antioquia Universidad de Antioquia Colombia angela.salazar@udea.edu.co

**Keywords:** perioperative period, nursing care, operating rooms, surgical procedures, operative., período perioperatorio, atención de enfermería, quirófanos, procedimientos quirúrgicos operativos, período perioperatório, cuidados de enfermagem, salas cirúrgicas, procedimentos cirúrgicos operatórios.

## Abstract

The study describes basic nursing care during the perioperative. Introduces the origins of perioperative nursing, general care that must be practiced with patient in this context. During the preoperative, care related with risk assessment and preparation of patient from the emotional and physical point of view are important. The trans-operative is related with the anesthesia used, surgical position, preparation of the skin, maintenance of normothermia, among many others. The postoperative depends on the type of anesthesia and surgical procedure, emphasizing on airway permeability, hemodynamic stability, pain, and symptomatology being presented by patients until they are stable and suitable for transfer to another service or their home.

## Resumen

Se describen los cuidados de enfermería básicos durante el perioperatorio. Se presentan los orígenes de la enfermería perioperatoria, los cuidados generales que se deben tener con el paciente en este contexto. En el preoperatorio es importante los cuidados relacionados con la valoración del riesgo, la preparación del paciente desde el punto de vista emocional y físico. Durante el transoperatorio se relacionan con la anestesia utilizada, la posición quirúrgica, la preparación de la piel, el mantenimiento de la normotermia, entre muchos otros. En el posoperatorio dependen del tipo de anestesia y procedimiento quirúrgico, realizando énfasis en la permeabilidad de la vía aérea, la estabilidad hemodinámica, el dolor, y la sintomatología que va presentando el paciente hasta que este estable y apto para trasladarse a otro servicio o para su casa.

## Resumo

São descritos os cuidados básicos de enfermagem durante o período perioperatório. São apresentadas as origens da enfermagem perioperatória, bem como os cuidados gerais que devem ser tomados com o paciente nesse contexto. No pré-operatório, são importantes os cuidados relacionados à avaliação de risco e o preparo do paciente do ponto de vista emocional e físico. Durante o transoperatório, estão relacionados à anestesia utilizada, à posição cirúrgica, ao preparo da pele, à manutenção da normotermia, entre muitos outros. No pós-operatório, dependem do tipo de anestesia e procedimento cirúrgico, enfatizando a permeabilidade da via aérea, estabilidade hemodinâmica, dor e os sintomas que o paciente apresenta até que esteja estável e apto a se transferir para outro serviço ou para sua casa.

## Introduction

Perioperative nursing, according to history, dates to 1873 in the United States when schools of care were created. As specialization, it was recognized prior to 1889,^1^ but the first reference of nurses in a surgical center is found in the appendix of the text Notes on Nursing by Florence Nightingale that states: “The surgical nurse must always be alert, always on guard, against the lack of cleanliness, musty air, lack of light…”.^2^ In 1978, the Association of Operating Room Nurses (AORN) introduced the definition of perioperative nursing, as the process of care during the perioperative, which is temporary and experienced by patients during the preoperative, trans-operative, and post-operative periods,^1^ a concept that cleared the path for nursing to areas different from the surgical center.

Today, the number and complexity of surgical procedures have increased due to progress in surgical, anesthetic, technological, and pharmacological techniques, along with changes in the offer of health services, which favors recovery and stabilization of patients, with greater control of pain, nausea and vomiting, which has reduced hospital stays, risk of infection, and costs.^3^ Most of the time, the postoperative takes place at home, which promotes outpatient surgery and self-care.^4^ Nursing, within this context, requires expert knowledge on guides and care standards related with surgery, anesthesia, invasive procedures, instrumental and surgical equipment, infections, and patient safety, among others. Likewise, nursing must have human quality, excellent interpersonal and social relations in the surgical context, besides leadership and good communication.^5^,^6^ In this regard, the Joint Commission International continues identifying errors in communication^7^ and has identified it as objectives in patient safety, given that work in this context is complex and errors in communication occur when the staff is under stress, or conducting multiple tasks and when there are interruptions in communication, which can interfere with the cognitive process.^8^ Moreover, the staff is prone to errors when there is no comprehension of their role, or do not feel supported, respected, valued, understood, or listened to, or does not have the training to provide high-quality information to their peers and patients.^9^,^10^ Communication affects patient satisfaction^11^ when they are not heard, or are interrupted, or when their beliefs or concerns are ignored. Additionally, if they perceive instructions difficult to follow or do not understand what they should do, it affects their compliance.^9^

Among the safety aspects included in the perioperative there is participation from the health staff and from the directors of the institution in patient safety in the surgical room. Nursing has led this initiative, but requires commitment from other professions. Establishing a culture of patient safety, defined as the “set of practices shared by the health staff and planned institutionally to avoid risks to patients during the pre-, intra- and post-operative periods”.^12^ Using surgery checklists ensures reducing damage to patients whether by adapting protocols to specificities, which must be in continuous improvement plan. Increasing the number of nurses in the surgical room to remain more time in the operations room, with greater autonomy in their work. Equilibrium of physical, material, and human resources necessary for the surgical procedure in amount and quality, with preventive maintenance plan. Technical-scientific updates through continuous education for health professionals. 

## Nursing care during the preoperative

This care starts from the moment patients decide to be operated and ends with their transfer to the surgical room. The surgeon explains the pathology, surgical approach, need for amputation, possible functional alterations; explains whether the surgery is urgent or not, the objectives, methods, expected benefits, potential dangers, inconvenience, probabilities of complication and makes a prognosis to make decisions.^13^ Patients must know their freedom to withdraw from the procedure if they wish. Patients are also seen in the pre-anesthetic consultation, which is a “clinical process that precedes the anesthetic-surgical event and consists in obtaining information from distinct sources (medical file, patient, results of laboratory exams, or others) to plan correctly the anesthetic and surgical procedure, besides offering guidance to patients and solving all their concerns”.^13^

The anesthesiologist must select the medications and doses indicated according with the physiological conditions and age of the patient. Nurses are responsible for their correct preparation and application. After assessing the patients, they are classified according to the following American Society of Anesthesiologists (ASA) physical status scale:^13^**“** *ASA I:* Healthy patient with localized disease; *ASA II:* Patient with disease independent of the cause originating the intervention; *ASA III:* Patient with severe systemic disease; *ASA IV:* Patient with severe incapacitating disease that conditions risk of death; *ASA V:* Moribund patient with life expectation < 24 h with or without surgery; *ASA VI:* Patient with brain death: organs will be donated”.

This phase of nursing care assesses the dimensions of the human being to identify problems of patients and their families, includes inquiring for antecedents and physical evaluation from which emerge actions that must have education as central to diminish anxious processes that promote post-operative recovery, besides reviewing presurgical laboratory tests and already known risk factors. During the evaluation, the peri-operatory nurse obtains important information to plan the care. This must start with identifying the patient and confirming the correct site of the surgery; reviewing the clinical chart including results of laboratory exams and other diagnoses. It is necessary to understand the disease process and the procedure to be performed and evaluate the emotional state.^14^

*Preoperatory preparation.* Surgery constitutes not only risk, given the possibility of infection and metabolic disorders, but also an emotional crisis for patients and their families, due to questions raised. Caring for children and adults includes the physical and emotional dimensions, mitigating fears of pain, death, bodily image, family separation etc. To solve these problems, it is necessary to hold preoperative meetings to facilitate anamnesis and know of doubts and concerns that can be related directly with the surgical experience.^15^ Attributes, like their listening capacity, fraternal impartial and friendly attitude stimulate trust and allow patients to familiarize with those who will care for them in the surgical room. The objectives are related with information about the trans-operative and post-operative, involving the patient and the family in the care.^15^ Surgical and anesthetic antecedents are evaluated, as well as the support the patient has and the possible necessities in the postoperative. This phase is an opportunity to work on educational issues.

Personal antecedents. It is important to know of allergies to medications or other agents, like antiseptics, foods; in case of any of them existing, this must be made visible to the patient and in the clinical chart, also, medications being taken (for diseases or through habit), including over-the-counter drugs, many are adjusted before the surgery, by the surgeon and anesthesiologist. Current state of comorbidities, like hypertension, diabetes, pulmonary status, and asthma. It is important to inquire about personal or family antecedents of bleeding and anesthetic problems, or possibility of anemia; with women, inquire about the pregnancy, and surgical history and trauma. Uncontrolled high blood pressure is a risk factor calling for patient stabilization before admission to the surgical room.^16^ If patients suffer ischemic myocardial disease, this must be studied prior to the procedure;^16^ likewise, assess if they have stents, their type and need for platelet anti-aggregation, and the cardiologist defines the situation to the patient.^17^

*Diabetes Mellitus.* If the patient is controlled, risks are similar to that of non-diabetic patients. The aim is to achieve concentrations between 140 and 180 mg/dl; in uncontrolled diabetics, complications range from infections and wound healing disorders to metabolic or hydric imbalances.^17^

*Respiratory disease.* Infections must be treated prior to surgery. Chronic obstructive or restrictive pulmonary diseases increase risks, require evaluation and treatment prior to going into surgery. Patients who smoke, ideally, must suspend tobacco use at least two weeks before the procedure to “permit recovery of the mucociliary transport mechanisms, reduce secretions, and lower carbon monoxide levels”.^16^ Asthmatic patients must have their disease controlled before the surgery. Training with breathing exercises is beneficial in obese patients with respiratory disease or those subjected to thoracic or abdominal procedures.

*Kidney disease.* In presence of nephritis, acute kidney failure, surgery is contraindicated. Chronic kidney failure is not a contraindication. If the glomerular filtration rate is < 30%, morbidity increases due to electrolyte disturbances, metabolic acidosis, high blood pressure, and uremic states that undermine life.^18^ Alcohol consumption induces reactions and tolerance against anesthetics, requiring greater doses of analgesics during the postoperative and monitoring for the possible onset of Delirium Tremens.^19^

*Risk of thrombo-embolic disease*. Formation of a thrombus in the venous vessels, due to prolonged rest and/or endothelial injury, causes local disturbances to obstructing pulmonary vessels.^20^

*Obesity.* Increases anesthetic risk and makes the surgical technique difficult, thus, it must be assessed carefully.^16^ Currently, presurgical exams are requested according with the patient’s specific conditions and clinical characteristics.^21^

Informed consents. These are related with the surgical procedure, conducted by the surgeon; the anesthetic, performed by the anesthesiologist; and nursing that is related with all the processes and procedures carried out by the nursing staff during the perioperative period, which is under the responsibility of nursing. The consent “is a legal instrument that defines the obligations of the parties involved in the act”. Once patients are informed and have their doubts cleared, they decide to accept or reject it; in this case, potential problems are informed and written record of their refusal is included in the clinical chart. This document is signed by the patient and the caregiver or companion who accepts said procedure.^22^

Physical preparation. It is necessary to bear in mind the following aspects in patients: 

*Preparation of the skin* **.** To suppress transient flora and inhibit resident flora, thus, eliminating one of the sources of surgical infection, This is achieved by bathing and asepsis of the surgical area.^23^ The presurgical bath is done with antiseptic soap, focusing on contaminated areas, like the navel, perineum, inguinal folds, and armpits, the previous day or the day of the intervention, including washing the hair with shampoo.^23^,^24^ If the procedure is in the head, two baths with 4% chlorhexidine are recommended.^23^

*Hair removal from the operative area.* The current trend consists of making a very sparse trichotomy, avoiding lacerations because they are infectious foci. It should be done with an electric razor, and disposable heads, or with depilatory cream, after testing for sensitivity,^25^,^26^ immediately before the surgery (2 h before) and outside the surgical room. Do not use adhesive bandages tape to remove the hair because it can cause microaggressions that permit colonization with bacteria; patients must be educated not to shave the surgical zone.^23^

*Preoperative fasting* **.** If the patient requires special preparation, the specifications appear in the medical orders. General anesthesia increases danger of bronchial aspiration when the patient is urgent and fasting time is unknown.^27^ In children, this risk is prevented by educating the family. Fasting of clear liquids must be of 2 h, breast milk 4 h, non-human infant formula 6 h, light food 6 h, and normal diet at least 8 h.^28^

*Hematic products:* in surgeries with prognosis of blood loss, the request must be verified with the blood bank and the patient must be classified with cross tests and sufficient reserve of hematic products.

*Dressing and preparation of the patient* **:** on arrival to the surgery service, street clothes are substituted by surgical gown, turban or disposable cap and surgical leggings. The patient is brought to the operating room without makeup, to be able to see the color of the integuments. All types of prosthetics, contact lenses, piercings and jewelry are removed. 

*Patency of venous line*: a good gauge catheter, minimum 18, is installed in the back of the hand and/or forearm away from flex sites to provide a pathway to administer medications and solutions. Likewise, the patient is weighed because drug doses depend on weight. 

*Preparation of the colon:* Elective surgery of the colon requires mechanical preparation. The objective is to arrive to surgery with an empty intestine and free of pathogenic germs. If required, it will be ordered by the treating surgeon for its preparation.^29^

*Safety indicators*: it is necessary to verify the patient, surgery, and correct side to prevent unwanted events. In addition, these are confronted with the clinical chart, surgery programming and other exams, like imaging, and other identification mechanisms.

*Education for the postoperative:* plays a very important role in the patient’s recovery and these must be taught breathing exercises, like coughing; active and passive exercises of upper and lower limbs; changes of position, early ambulation, management of catheters and drains, how to get up, among many others. The educational interventions have positive effects on knowledge, satisfaction, physical, mental, and social aspects, quality of life, knowledge of self-care practices.^30^

Emotional preparation. It helps to minimize negative emotions related with the surgery; ideally, this starts three weeks before such. The surgical intervention is a trigger and stimulus that impacts the human being’s emotional dimension and can bring unpleasant consequences that can lead to preoperative trauma.^31^ For some patients, surgery can cause concern and anxiety throughout the perioperative process, in part, due to lack of experience with surgical procedures. Anxiety can also be based on the fear of pain and the anesthesia.^32^ Lack of information, not only of the surgical procedure, but of the anesthesia and its complications, fear of the side effects, and the potential risk of death are aspects that can trigger anxiety.^33^

Anxiety is manifested with increased state of alertness, heart rate, blood pressure, muscle tension, and respiratory disorders.^34^ Superficial bodily signs may include pale skin, sweating, shivering, and dilated pupils.^35^,^36^ The surgical intervention reactivates memories of traumatic situations, previous personal surgical experiences or of significant relatives that can represent a stressful event and generate concerns, like death, physical dependence, not returning from the anesthesia, pain, disease, recovery and separation from the family generate emotional responses, like anxiety, depression and stress that make the post-operatory recovery much slower and complicated. Hence, preparing the patients and providing them with information, will allow them to understand what they expect to find during the perioperative period.^33^

Tranquility can also be manifested and this is because their body and their environment are not governed by themselves, rather, there is a spiritual or material period that governs them, which can be called destiny, God, supernatural, or whatever.^32^,^37^ That period “is manifested in the hands of the surgeon and the accompanying staff. Prayer or profound dialogue with God It is to request and entrust that everything turns out well, since it is from Him that everything is expected, in Whom one trusts and to Whom life is given”.^38^,^39^ “It is through spirituality that they find hope, relief and inner peace during this process; these findings are compatible with other studies that examined the association among religious participation, spirituality and health and found that religious participation and spirituality are associated with better health results”.^40^^-^42 The person entering surgery must confront multiple stressful elements, like personal, physiological, psychological, and environmental factors.^41^

Emotional needs are determined by the capacity to adapt to situations that represent danger, fear, and anxiety and every emotional adaptation process demands a process of information.^42^ Psychological support is quite important, it is necessary to banish the ghost of bad luck or the idea that something bad will happen. When things are done right, good indication and preparation, adequate surgical technique, the end result of an operation should be favorable. Hence, the following should be considered:^43^

*Information:* education/teaching the patient reduces pre-operatory anxiety. Explaining the procedures and activities of nursing care and the feelings they will experience in the pre-, intra- and post-operative. 

*Psychosocial support:* interactions intensify behavior mechanisms related with anxiety and fears and provide emotional wellbeing. 

*Training of skills* **:** practice guided by specific measures makes the post-operative period simpler, accelerates recovery, and helps to prevent complications.

In addition, emotional care includes physical presence that implies listening and explaining; physical contact that expresses feelings of comprehension, interest, trust and significance of active presence; visual contact during verbal or non-verbal communication; providing guidance to promote self-care; assisting with physical presence and empathic attitude in situations of explicit or implicit vulnerability, both of the patient and of the relative; providing interventions to potentiate control of thought processes so that negative beliefs are substituted for positive attitudes; capacity to understand and respond affectively and adequately to the physical, emotional, and spiritual needs of the patient, expressed verbally and non-verbally; keeping adequate distance and providing trust and intimacy for patients to express themselves; providing adequate and individualized information according to the patient’s and family’s situation; facilitating visits from a priest, chaplain or pastor according to their religion.^44^ Patients must demonstrate they understand the surgical experience and must receive pre-operatory education and for the post-operative; knowing the time of the surgery; knowing the post-operatory unit and the location of the family during the surgical intervention and after recovery; knowing the monitorization and treatments foreseen in the post-operative; knowing the resumption of their activities and the measures to relieve pain, as well as permitting to express their feelings regarding the surgery. 

## Nursing care in the trans-operative

Within the surgical context, care is offered with high technology, thus, it is essential to have good knowledge of medical devices. The operating table is narrow and through its sections, it can be adapted to the surgical procedure and the patient’s conditions. This is located in the center, under a ventilation and illumination system in the operations room.^45^ The purpose of the ventilation system is to keep temperature from 18 to 25 °C with humidity > 50%, and carry out 25 air replacements per hour to reduce the air’s microbial content and dilute anesthetic gases.^46^ Noise is common in the surgical room due to medical equipment (surgical drills, hammers, among others), telephones, alarms, music, intra-professional communication.^14^

The surgical team is comprised of members from different professions, like specialists in anesthesiology, surgery; circulating and instrumental staff. The circulating staff can be nurse specialist in surgical care, nurse, or nursing aide.^47^ All work together to perform the surgical procedure and optimize patient care. The care provided in the surgical room is characterized by efficiency exigencies;^48^ organizational barriers and shortage of staff hinder contact with patients, satisfying their needs and expectations, making it necessary to adapt the care to the patient’s conditions during the short time prior to the anesthesia or sedation.^49^

Care in this context should be centered on the person, seeing the human being as an independent person, with dignity and in need, emphasizes the individual will and abilities of the person.^50^ Various studies describe the surgery staff as healers of the patient’s anxiety during a surgical procedure^51^^-^54 by perceiving them as serious and competent, which generates trust and tranquility in patients.^54^ The staff’s attitude affects the patient’s concerns and anxiety, intraoperative soothing properties took place when the staff had a positive and friendly attitude in dealing with the patient and among them. 

In this phase, there is disturbing commotion that generates anxiety for patients upon seeing the physicians in surgery, noting the instruments with which they will be operated or observing any surgical compress or bloody gauze. This anxiety can determine the need for more anesthesia and, hence, risks. Patients awake during the surgical procedure hear everything in the surgical room, such as conversations by the staff and the noise related to the procedure that can be annoying.^32^ A high level of noise in the surgical room can lead to post-operative sleep disorders. Anxiety experience throughout the process can provoke increased post-operative pain, nausea and vomiting, as well as delayed post-operatory recovery and hospitalization. Post-operative pain increases because anxiety creates diminished tolerance and pain threshold, which - in turn - prolongs the patient’s post-operatory recovery in hospital or other care center.^35^^-^55

Touch and contact have been shown to reduce anxiety^24^,^25^,^54^^-^57 through massage, or by holding their hands, besides being described as anxiolytic, and diminishes systolic blood pressure.^52^ Anti-stress balls also help, which can be squeezed in case of perceived concern or anxiety.^51^ Part of the patient’s comfort in surgery is that of having a heating blanket; it contributes to feeling that the surgical team prioritizes the patient’s physical comfort and the heat contributes to relaxation that relieves anxiety.^54^ Sound distractions during surgery have been used and patients appreciate such positively; these relieve anxiety and aid in normal heart rate,^58^,^59^ blood pressure, and respiratory frequency.^54^,^58^,^59^

Patient’s preparation in the surgical room. The surgical team: instrumentation, circulating staff and physicians, simultaneously, work on preparing the patient for the surgical event, again identifying the patient, confirming the surgery: patient, procedure and site of intervention; position in the surgical table, monitoring physiological constants: heart rate electrocardiographic record, non-invasive blood pressure, pulse oximetry, capnography continuously to spot disorders, bearing in mind that no monitoring apparatus substitutes clinical judgement. Other preparations include preservation of normothermia, taking precautions to avoid deep venous thrombosis, as well as disinfecting the feet^60^ among many others, which represents exposure and manipulation of the patient’s body. Prior to starting any surgical procedure, it must be ensured that all the anesthetic and surgical material and equipment are available for the surgical event. 

Anesthesia. For the procedure, patients must be subjected to some type of anesthesia: general, local or regional. With general anesthesia, the patient is unconscious through the administration of different types of medications, anesthetics, analgesics and, if necessary, muscle relaxants. Local anesthesia is used to anesthetize a small area of the body, while with regional anesthesia the body area is broader. With local and regional anesthesia, the patient is awake during the surgical procedure, probably sedated.^61^ Activities are aimed at protecting the patients’ safety and wellbeing, like their necessities through monitoring the activities of the members of the surgical team and constant revision of prevalent conditions in the surgical room: appropriate asepsis, temperature, humidity, and lighting, availability and proper functioning of equipment and instrumentation.

Venous and arterial lines. The patient’s characteristics and comorbidities, the surgical procedure and possible recovery in the intensive care unit require continuous invasive monitoring of the patient, given that it allows observing physiological changes. Arterial catheterization allows continuous monitoring of systolic, diastolic and mean arterial pressure (MAP);^62^ also, central venous catheter to monitor central venous pressure, fluids in large volumes, administration of medications, insertion of catheters in the pulmonary artery, no peripheral access, among many others.^63^ Generally, once the patient has been anesthetized, catheters are inserted after skin asepsis in the corresponding areas. 

Surgical positions. The objective is to obtain an optimal exposure of the region to be operated, access to venous catheters, and monitoring devices monitorization. Attention should be paid to the patient’s comfort and safety, as well as to the circulatory, respiratory, musculoskeletal and neurological structures. It must be a time of great attention, being frequently performed routinely and often underestimated. The body in a certain position exerts external pressure on the patient's tissue that, at capillary pressures > 32 mm Hg, causes occlusion of blood flow that inhibits tissue perfusion and produces tissue ischemia.^64^ It is recognized that 23% of intraoperative pressure lesions are acquired in procedures lasting more than three hours;^64^ besides, patients remain still during the surgery and cannot change position or feel pain caused by remaining in a position during a prolonged period or even manifest verbally their discomfort in a given position. Patients under local anesthesia might not feel pain or might not be able to communicate where the pain is felt;^65^ thereby, they depend on nursing and members of the surgical team to advocate for them. Many times, with a given position, uniform distribution of body weight is not allowed, which leads to the risk of tissue damage. Skin areas over bony prominences are particularly vulnerable to pressure lesions (PL), above all in individuals with low weight; this is why risks must be identified and start their prevention.^65^

The procedure and status of the patient determine the equipment to use to provide the position to the patient; the staff should check that the surgical table has all its accessories and performs all its movements. Once the patient is anesthetized, said patient is placed in the position required for the surgical procedure, prior identification of potential risks evaluating the patient’s needs and characteristics, like weight, height, and age.^66^ The most common surgical positions are dorsal decubitus, Trendelenburg, reverse Trendelenburg, batrachian, lithotomy, prone position, Kraske (jackknife), lateral decubitus, Fowler or semi-Fowler. Each position produces physiological and anatomical alterations and can produce pressure zones.^67^

Avoiding pressure lesions during the intra-operatory: surgical patients are vulnerable to their development as a result of multiple risk factors:

*Intrinsic to the patient:* the skin’s capacity against pressure and cutting and shearing forces, adjusted to old age; medications; comorbidities, like cancer, vascular or cardiovascular disease and diabetes mellitus; low body mass index; low systemic blood pressure; low hemoglobin and hematocrit levels; poor nutritional state with low albumin levels; and diminished blood pressure.^68^

*Extrinsic to the patient:* the conditions of procedures performed to solve a condition through a surgical intervention^69^ depend on physical and environmental factors and include shearing, friction, humidity, position and duration of the surgery. Often, these lesions are not present in the immediate post-operatory and can take up to five days to become visible.^65^ Foam-based materials, specifically D33 sealed foam, redistribute the body interface pressure on operating tables more effectively.^70^

Surgical site infection (SII), generates great consternation for patients and relatives and as a high cost for health systems. Thereby, its prevention must be a priority for everyone. The risk factors that can cause it are related with prolonged hospital stay before or after the surgery, unsubstantiated prescription of antimicrobials, deficient antiseptic cleaning of the patient’s skin prior to the surgery, and others oversights, like lack of hand hygiene.^71^ Likewise, intrinsic risks exist related with patients, like their comorbidity diseases, nutritional state, smoking, obesity, and aging.^72^ Considering that a patient with SSI has five more times the risk of dying than an uninfected patient, and his/her additional care generates costs close to 2,625 US Dollars, coupled with the loss of expectation of health, surveillance, prevention, and control strategies result necessary in this type of infection associated to healthcare.^73^

Prophylactic antibiotics. Risk of infection depends on the magnitude of the wound’s contamination wound and the host’s resistance. Efforts are made to control the sterile technique, but host and procedural factors make it a problem, so each hospital dictates the rules on the subject; the prophylactic antibiotic is selected according to spectrum, pharmacokinetics, toxicity, frequency of adverse secretions and the possibility of achieving good concentrations in a single dose, and costs. The most-used are cephalosporins. Their effectiveness depends on the moment of administration and on the time maintained. It should be administered 1 h before the surgical event and more than 24 to 48 h is not justified.^73^ The infections committee at each hospital formulates its own protocols and methods to carry out the practice, adjusted to economic resources and characteristics of the population.^74^

Preparation of the skin for surgery. Before starting antisepsis, there is surgical hand washing for 2 to 5 minutes, as recommended by the World Health Organization, 75 and use of sterile gloves. At the same time, the skin is examined to identify the presence of organic matter or dirt (this must be removed with gauze and non-sterile gloves) and report and register any alteration, like nevi, warts or other. The effectiveness of antiseptics depends on the skin being clean, free of organic material and waste. In preparing the skin, emphasis is placed on the most contaminated areas: navel, armpits, folds, subungual region, foreskin, and others. Preparation of clean and contaminated areas for the procedure is performed separately to avoid microorganisms in the incision site^23^,^24^, like when there are stomas. Remember that povidone iodine is inactivated by organic material.^23^ Likewise, before starting the preparation of the skin, verify the surgical site to avoid preparing an area and performing the surgery on a mistaken site.^23^ Different antiseptic products have been used, these should not be irritating, but of broad spectrum, fast action, and residual effect. Antiseptics, like chlorhexidine should not be applied in the auricle and ear canal; in mucous membranes, the concentration decreases.^23^,^24^ Each institution creates its own policies for the antiseptics used, their concentrations and areas where these are used.

Upon ending the antisepsis, the sheets and equipment to position the patient must be protected from the antiseptic soaps used, the zone must be dry and the electrodes must not have direct contact with antiseptics to avoid reactions or adverse events in the patient.^14^,^23^ Usually, asepsis is carried out with sterile gloves to apply the antiseptic, unless it has a long device that does not permit contact of the non-sterile glove with the skin. It is administered by painting or rubbing the skin, no advantage has been reported of any of the methods; the time of application depends on the manufacturer, times range from 30 to 120 seconds.^14^,^23^

If the area is clean, start from the incision site towards the periphery in circular manner with increasingly larger circles. In many surgical procedures, the incision site is adjacent to contaminated areas and performing the asepsis of the incision site towards the periphery, avoids contamination from these areas to the surgical site. In case the procedure involves the penis, the foreskin must be retracted and asepsised; then put into place to avoid vascular alterations.^14^,^23^,^76^ When there is a contaminated area adjacent to the surgical site, without it being part of it, it can be isolated with adhesive or fluid-resistant tape.^14^,^23^

Gauzes, sponges, or applicators are used only once to avoid contamination of the incision site. A norm of asepsis in preparing the skin is to never proceed from a clean area to a contaminated area; hence, if preparing a contaminated area, prepare first the area with lower bacterial count and then the contaminated area; the technique would be from the periphery towards the incision site that is contaminated.^10^,^23^ If using a commercial applicator, follow the manufacturer’s instructions for its use. Upon ending the preparation, make sure the patient is on a dry surface.^23^ Traumatic open wounds are irrigated with saline solution.^23^ When requiring asepsis of vagina in abdominal procedures, splashing from the vagina on the abdominal wall should be avoided.^14^,^23^

Inadvertent hypothermia. It is defined as temperature < 36 °C during surgery; it is a preventable surgical complication.^77^ Studies indicate incidence between 11.7% and 94.4%.^77^^-^83 Hypothermia is associated with the alteration of the metabolism of medications,^84^ infection of the surgical site,^85^ paralytic ileus^86^ and post-operative cardiovascular events,^79^ increased risk of bleeding,^87^ increased consumption of red blood cells,^88^^-^90 changes in platelet function, increased oxygen demand accompanied by shivering; besides, greater use of intensive care, hospitalizations and long stays in recovery.^77^,^88^,^90^ Hypothermia is related with the anesthetic-surgical procedure, as with the temperature of the surgical room, duration of the surgery, administration of cold venous fluids, exposure of the bodily surface, loss of fluids and blood. It is also related with factors intrinsic to the patient, like age, sex, systemic disorders, and body mass index.^78^,^91^ In spite of the recommendations for maintenance of normothermia in the perioperative, this practice continues being a challenge to health professionals;^92^ in Colombia, this monitoring is conducted only in 10% of surgical patients,^93^ which is why interventions in hypothermia management must be a priority to guarantee safe and quality care to surgical patients.^94^ The results of the study by Zaman *et al*., conclude that using a warm solution (38 ℃), rather than a solution at room temperature, can prevent hypothermia and reduce post-anesthetic chills in patients subjected to abdominal surgery.^95^

Gastric emptying. The nasogastric tube is frequently used in surgeries of the upper abdomen; in cases in which it is necessary to decompress the stomach and evacuate the fluids contained in it, the Levin catheter must be installed.

Urinary catheter. Although patients are asked to empty their bladder prior to surgery, on special cases it is necessary to install a urinary catheter as prevention of bladder distention in surgeries of the lower half of the abdomen in which a full bladder would be a mechanical obstacle for the intervention in cases in which monitoring urinary output is desired and to ensure or control the flow of urine in urological surgery. The most used is the Foley catheter and it is connected to a collection bag. Generally, it is installed when the patient is under anesthesia and ingestion and elimination control is carried out by schedule.

## Nursing care during the post-operative

The challenge of post-anesthetic care with an increasingly aging population is the management of chronic diseases, health conditions, and progress in surgical interventions; increasingly, greater numbers of patients are attended with multiple comorbidities for numerous complex surgeries.^96^ The recovery or post-anesthetic period starts when the patient is transferred to the recovery ward or post-anesthetic care unit (PACU) until the patient recovers conscience and capacity to communicate, maintaining, protecting and stabilizing their respiratory tract and achieving cardiovascular health. This period is not free of risks, hence, patients subjected to neuroaxial or general anesthesia must be cared for in specially designed areas and staffed;^96^ it is a clinical area designed within the surgical center in which patients receive continuous care after surgery and anesthesia. These areas are served by trained individuals who provide care to patients until they are in conditions for discharge or are transferred to other clinical areas of the institution.^96^ An anesthesiologist must be available at all times to assess and manage patients whenever clinical necessity arises, or on emergency call. 

Continuous professional development is necessary to maintain standards and guarantee that knowledge, skills and update of new techniques and progress are obtained, such as management of difficult airway and pain management with pharmacological and non-pharmacological means. Basic competencies for care in this unit are related to professional ones, such as communication, professional development, clinical leadership. Clinical skills relate to evaluation and management of respiratory tract; circulation, conscience, monitoring during the immediate post-operatory phase, intravenous access and balance of fluids, knowledge of pharmacology applied to peri-operative care, management of post-operative pain, nausea and vomiting and of surgical and anesthetic emergencies. Skills simulations are means through which reanimation, algorithms, and management of anesthetic and surgical emergencies can be rehearsed. These need not be of high fidelity and permit formation of the multidisciplinary team.^96^ Patient care uses different empirical indicators, which include the Aldrete scale and the Bromage scale among others. The unit must be equipped with minimum pulse oximetry equipment, multiparameter monitors that display the electrocardiogram, non-invasive pressure, capnographs in case the patient is intubated, thermometers, crash cart, defibrillator, infusion pumps, among many others.^96^

Transition of patient from surgical room to recovery. An integral part of the continuity of good quality care is the effective transfer of clinical information. During the peri-operative, the patient goes through multiple processes: pre-operative, intra-operatory, post-anesthetic care unit and, finally, discharge to another service in the institution.^96^ Anxiety is also present in the post-operative, Pezzim *et al*., identified that patients with prevalent symptoms of anxiety and depression are more dependent on nursing care than asymptomatic patients.^97^

Transference of information between the surgical team in the surgical room and nursing in the post-anesthetic unit starts once the patient’s stability has been monitored and confirmed, using the suggested SBAR form (*situation, background, assessment and recommendation*)^96^: *Situation:* name and age of patient, operation practiced; *Antecedents:* past medical history, allergies to medications, anesthetic technique (including management of respiratory tract, analgesia, antiemetics, and intravenous fluids administered), any surgical intra-operatory event or significant anesthetic, or complications (like difficult airway, blood loss, cardiovascular instability, etc.,); *Evaluation* **:** airway: permeability, device *in situ*, possible difficulties anticipated; breathing: oxygen requirement, respiratory frequency, need for capnography; circulation: stability, presence of vasoactive infusion, invasive monitoring; and: *Recommendations:* requirement of continuous monitoring: type and duration required, analgesic plan, antiemesis, management of fluids: oral intake or requirement of intravenous fluids, investigations required: blood analysis, like hemogram, etc., additional information: drains, special dressings.

Clinical challenges in post-anesthetic care. The following are among the most important:

*Respiratory*. For discharge from the recovery service, patients must be conscious, with unscathed reflexes and able to maintain their own respiratory tract, permeable airway and respiratory frequency. Obstruction of the respiratory tract can occur at any moment with repercussions, like pulmonary edema and hypoxemia.^96^ Many patients arrive to the unit with a Guedell cannula or orotracheal tube; in this case, connection should be conducted to the capnograph for early detection of respiratory tract obstruction, as well as hypoventilation that results in hypercapnia. The staff must be trained in the extraction of said devices in the respiratory tract. In the case of an endotracheal tube, the responsibility for its removal rests with the anesthesiologist or such delegates this function on trained staff prepared to assume this responsibility. It is recommended to draw up a respiratory tract care plan during the delivery of patients to the staff, in patients with risk of airway complications or with difficult respiratory tract, equipment and experienced staff must be available.^98^ Post-operatory pulmonary complications can happen to 20% of the patients undergoing an important surgery.^99^ Early recognition of complications can minimize such by monitoring the respiratory frequency, pulse oximetry, and capnography under the circumstances described, as well as administration of oxygen.^96^ It is also important to know the morbidity of postoperative residual curarization, which is why the nerve stimulator can be available to evaluate the residual block when clinically suspected in patients who have received neuromuscular blocking drugs.^96^

*Cardiovascular.* The cardio-depressant effects of residual anesthetic agents, as well the loss of perioperative blood and changes of fluids, make patients prone to potential cardiovascular instability. Along with oxygen saturation monitoring, electrocardiographic tracing and non-invasive blood pressure measurement are minimum follow-up standards, but many patients enter the recovery ward with invasive blood pressure and central venous pressure monitoring or of other line *in situ*, which is why the staff must be trained in their use, cardiovascular stability must be achieved to comply with discharge criteria. Individualized care plans are drawn up for each patient and every intervention needs to be documented, like post-operative fluids, medications, oxygen administered and if the administration of intravenous fluids continues, it must be prescribed prior to their transfer to another service.^96^

*Post-operative nausea and vomiting* **.** It is still a common and unpleasant experience for a third of patients and it is associated with prolonged stay in recovery and unforeseen readmissions.^100^ Protocols for antiemetics can be developed in the unit and patients should not be discharged until the inconveniences of nausea and vomiting are adequately controlled.^96^ Other inconveniences reported by patients are thirst, and as its intensity increases, the resulting discomfort is greater. Thirst should be evaluated by the health staff, so that it is adequately treated. It is expected that the evaluation will allow some reflections on the behaviors to assume during the recovery from anesthesia, with aim of improving care and humanization of caring for surgical patients.^101^

*Pain management*. A broad range of pharmacological and non-pharmacological strategies can be used to achieve optimal management of pain during the postoperative. Epidural-type neuraxial block of peripheral nerves can be used, as well as patient-controlled analgesia, music therapy, relaxation, touch, among others. The staff must be trained to manage these patients and recognize potential side effects derived from these techniques. Specific protocols for the administration of opioid analgesia must be in place to allow timely management of postoperative pain;^96^ music has had positive effects in pain management as non-pharmacological treatment; besides producing relaxation, distraction, and tranquility.^102^

*Specific groups of patients*. Patients subjected to general anesthesia or neuraxial block must receive the same standards of care described previously.^96^ Children must be cared for in recovery areas designed and with staff for pediatric population whenever possible. Specific teams, protocols, and algorithms for pediatric care must be available. These patients have to receive the same level of nursing care.^96^ During this phase of the post-operative, it is important to have in mind the relatives who are in the surgical center’s waiting area because this can contribute with the emergence of feelings, like anxiety, nervousness and, consequentially, stress. It is advisable to include the relative in nursing care, which includes providing information, dialogue, and respect.^103^

Discharge from the recovery service. Discharge is ordered when the patient has fulfilled the following criteria:^96^ recovery of protective airway reflexes and sustained airway; respiratory frequency from 10 to 20 and regular, SpO_2_ 96% or equal to the preoperative level, oxygen prescribed where indicated. Stable blood pressure and heart rate, values depend on pre-operatory measurements. No inexplicable or uncontrolled arrythmias. Level of conscience: pre-operatory orientation achieved or additional evaluation conducted. Pain: controlled and postoperative analgesia prescribed; nausea and vomiting under controlled treatment and prescribed when indicated. Temperature: postpone discharge as long as the central temperature is not at 36 °C. Wound/drains/dressings: intact dressings and without evidence of excessive blood loss from the wound site or drains. Neuraxial block: spinal <T6 or epidural sensory level, sensory level at or below the level specified by the anesthesiologist. Venous access: catheter without residual medications and permeable. Medications/intravenous fluids: infusions prescribed, controlled, and dully labeled. During the perioperative, what people value is to feel safe and relational aspects, like the explanations they receive and the treatment by the professionals.^104^

Record of nursing care in the peri-operatory. Every intervention and interaction conducted by nursing must be duly documented in the clinical chart and in the different records in accordance with the institution’s norms.

## Conclusion

Care during the peri-operatory is a complex process of relationships, with and for human beings: patients and health staff. Patients transit from the pre-operative to the post-operative, placing them at risk of adverse events; therefore, within this context, patient safety is present in every care provided. Hence, nursing must be aware of details in care so that the passage of patients through this context is as beneficial as possible.
